# The Charophytes (Characeae, Charophyceae) of the Caucasus

**DOI:** 10.3390/plants14121788

**Published:** 2025-06-11

**Authors:** Roman E. Romanov, Liubov V. Zhakova, Andrey N. Efremov, Galina Yu. Konechnaya, Olga N. Boldina, Dmitry F. Afanasyev, Tatiana V. Akatova, Denis G. Melnikov

**Affiliations:** 1 Dobra Voda, 85356 Bar, Montenegro; 2 Komarov Botanical Institute of the Russian Academy of Sciences, Professora Popova Str., 2, 197376 St. Petersburg, Russia; luba_zhakova@mail.ru (L.V.Z.); galina_konechna@mail.ru (G.Y.K.); boldina@binran.ru (O.N.B.); dmelnikov@binran.ru (D.G.M.); 3 Zoological Institute of the Russian Academy of Sciences, University Embankment, 1, 199034 St. Petersburg, Russia; 4 Research Center for Fundamental and Applied Problems of Bioecology and Biotechnology, Ulyanovsk State Pedagogical University, Lenin Sq., 4/5, 432071 Ulyanovsk, Russia; stratiotes@yandex.ru; 5 Russian Federal Research Institute of Fisheries and Oceanography, Okruzhnoy Proezd, 19, 105187 Moscow, Russia; dafanas@mail.ru; 6 The Faculty of Bioengineering and Veterinary Medicine, Don State Technical University, Gagarin Sq., 1, 344000 Rostov-on-Don, Russia; 7 Caucasian State Nature Biosphere Reserve, Sovetskaya Str., 187, 385000 Maykop, Russia; hookeria@mail.ru

**Keywords:** Characeae, *Chara*, distribution, flora, *Lamprothamnium*, *Nitella*, *Nitellopsis*, *Sphaerochara*, stoneworts, *Tolypella*

## Abstract

This first inventory of the charophytes of the Caucasus region was compiled based on records from published references, online sources, a review of herbarium collections, and our own field collections. The documented Caucasian charophyte flora includes 27 species from six genera: 18 *Chara* species, 6 *Nitella*, 2 *Tolypella*, and 1 species each of *Lamprothamnium*, *Nitellopsis*, and *Sphaerochara*. *Chara uzbekistanica*, *C. virgata*, and *C. contraria* var. *hispidula* are newly recorded for the Caucasus. The high species richness of the genus *Chara*, the much less diverse genus *Nitella*, and a few species of *Tolypella* and *Sphaerochara* in the Caucasian charophyte flora are typical traits of Palearctic charophyte floras. In total, there are 10 species recorded in Armenia, 16 in Azerbaijan, 18 in Georgia, and 16 in the mountainous region of the North Caucasian Federal District of Russia. Most of the species have wide distributions; none are endemic to the Caucasus. One of the most commonly recorded species in the region, *C. gymnophylla*, is a usual feature of the Mediterranean and West Asia. The Caucasian charophyte flora can be described as unsurprising from a large-scale perspective, considering its species distribution ranges. However, the association of species makes the region specific at the scale of West Asia when comparing it to its large neighboring areas.

## 1. Introduction

The charophytes (Charophyta, Characeae) are a charismatic group of macroscopic algae, easily recognizable because of their typical arrangement of thalli, consisting of repeating parts. They are widely known as bioindicators of water quality [1]; pioneer species of newly appearing water bodies; or as perennial species forming long-living stable stands, creating and maintaining highly peculiar environments for other aquatic organisms [2,3]. Their extensive communities can be keystone ecosystem engineers, resulting in high water quality. Recent studies have examined charophytes in many regions where the charophyte floras were poorly known [4,5,6,7,8,9,10,11,12,13,14,15,16,17]. However, in many regions the charophyte floras remain largely undocumented.

Charophytes are the most abundant submersed macrophytes in some ecosystems of the Caucasus, notably the lakes Sevan and Göygöl, the Sudzhuk Lagoon in the Krasnodar Territory, and Lake Zerik-köl in the Kabardino-Balkarian Republic [18,19,20,21,22,23,24,25,26,27,28,29,30,31,32,33]. However, only mostly scattered records of charophyte species in the Caucasus were available for a long time, leaving large knowledge gaps about the regional occurrence of species in time and space. The references covering more than one country [34,35,36,37] list records from the 19th to the first third of the 20th century, with a maximum of 46 geo-referenceable species’ distributional records in a single reference [37], a small number for this vast area. A species list and synopsis of the charophyte sites was compiled for Georgia, with 23 species’ distribution records, covered nearly all sites known until the second half of the 20th century [38]. All other Caucasian regions were without them until a recent preliminary compilation [14]. Records for even generalist species were lacking for many regions of the Caucasus, representing one of the most notable gaps. The preliminary generalization [14] suggested the absence of key and endemic species in the Caucasus, probably representing a crossroad for charophyte species with different distribution areas. In addition to the cosmopolitan and widely distributed species, the charophyte flora of the Caucasus consists of species with distribution areas mainly in inland Eurasia and in the Mediterranean and West Asia [14]. Testing the hypothesis about the low distinctiveness of the Caucasian charophyte flora requires a much more detailed dataset. Therefore, we aimed to compile all the distributional data for the Caucasus based on the existing specimens and all the available sources in an attempt to shed more light on the traits of the charophyte flora of the Caucasus, and to form a species list, a synopsis of the sites based on the specimens checked, and a summary tracking the changes in species occurrence, to stimulate charophyte research in this region. We dedicate this article to Anders Langangen (1942–2025), whose articles on charophytes from different regions of the world inspired us.

## 2. Results

**Figure 1:**
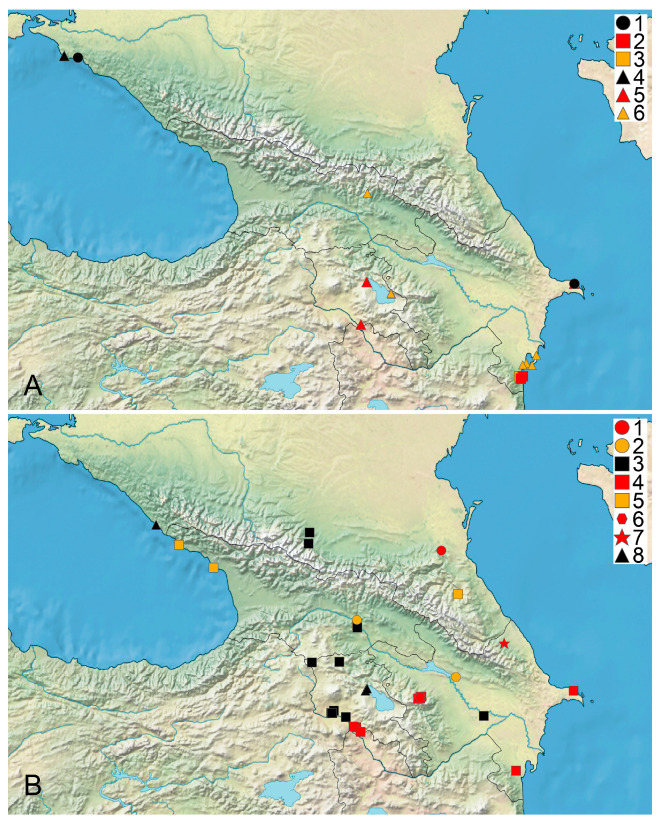
Distribution of charophyte species in Caucasus. (**A**) 1—*Chara baltica*; 2, 3—*C. braunii*; 4–6—*C. canescens*. (**B**) 1, 2—*C. connivens*; 3–5—*C. contraria* var. *contraria*; 6—*C. contraria* var. *hispidula*; 7—*C. denudata*; 8—*C. tomentosa*. Black marks are records from 1980 onwards, red marks indicate last recorded between 1950 and 1979, and yellow marks indicate last recorded before 1950 [39].

**Figure 2:**
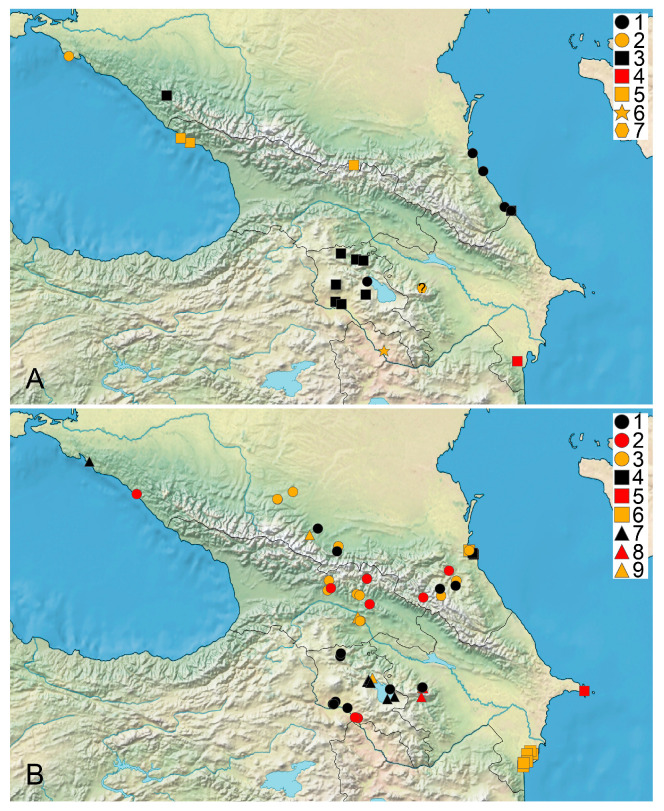
Distribution of charophyte species in Caucasus. (**A**) 1, 2—*Chara globata*; 3–5—*C. globularis*; 6—*C. hispida*; 7—*C. strigosa*. (**B**) 1–3—*C. gymnophylla*; 4–6—*C. neglecta*; 7–9—*C. papillosa*. Black marks are records from 1980 onwards, red marks indicate last recorded between 1950 and 1979, and yellow marks indicate last recorded before 1950 [39]; published record requiring confirmation is marked with “?” superimposed.

**Figure 3:**
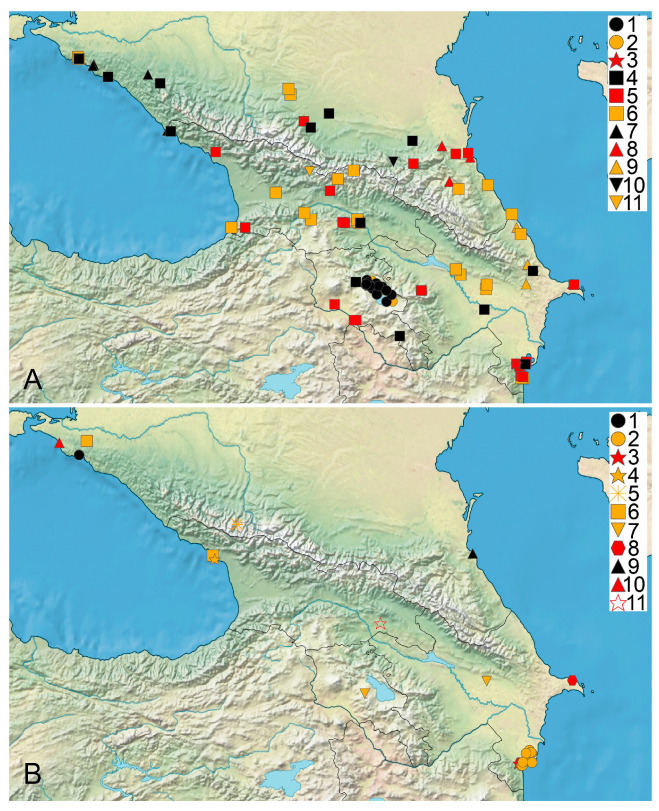
Distribution of charophyte species in Caucasus. (**A**) 1, 2—*Chara uzbekistanica*; 3—*C. virgata*; 4–6—*C. vulgaris* f. *vulgaris*; 7–9—*C. vulgaris* f. *longibracteata*; 10, 11—*C. squamosa*. (**B**) 1, 2—*Lamprothamnium papulosum*; 3, 4—*Nitella capillaris*; 5—*N. flexilis*; 6—*N. mucronata*; 7—*Nitella* spp.; 8—*Tolypella glomerata*; 9, 10—*T. nidifica*; 11—*Sphaerochara prolifera*. Black marks are records from 1980 onwards, red marks indicate last recorded between 1950 and 1979, and yellow marks indicate last recorded before 1950 [39].

**Figure 4:**
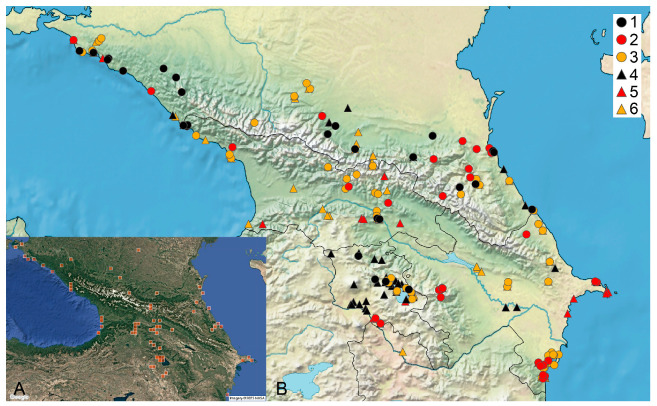
The distribution of charophytes in the Caucasus. (**A**) All recent records from iNaturalist.org [83]; a few records from outside of the studied area are visible too. (**B**) Records of charophyte species and unidentified charophytes according to the published sources and studied specimens; this excludes most records from iNaturalist.org, whose vouchers were not studied; 1–3—studied specimens; 4–6—published records. Black marks are records from 1980 onwards, red marks indicate last recorded between 1950 and 1979, and yellow marks indicate last recorded before 1950 [39].

The distribution data for, and the habitat and floristic novelty of, the species found in the studied area are listed below. The synonyms for the species reported under these names are indicated. The original labels in Russian are transliterated and translated, and their current toponyms are added in brackets whenever possible. The habitats are listed according to the specimens studied and the printed records. A long list of the sites of the generalist species are available in Appendix A. Time intervals at maps (Figure 1, Figure 2, Figure 3 and Figure 4) follow [39].

*Chara baltica* (Hartm.) Bruz. (Figure 1A)

Material examined: Russia • Krasnodar Territory, Black Sea, Novorossiysk, Sudzhuk Lagoon; 20 July 1925; A.D. Zinova, V.S. Stefanov leg.; LE. • Krasnodar Territory, Black Sea, Novorossiysk, Sudzhuk Lagoon; 21 September 1953; A.D. Zinova, V.S. Stefanov; LE.

Published records: Azerbaijan [40], Russia [41]. Their vouchers stored in LE and ROST were checked.

Habitat: Permanent brackish coastal lakes and lagoons.

*Chara braunii* C.C. Gmelin (Figure 1A)

=*C. coronata* Ziz ex Bisch.

Material examined: Azerbaijan • Lenkoran District, village of Sutamurdov [Lankaran District, Sütəmurdov (also, Sutamurdo and Sutamurdob)], in paddy fields; 9 July 1956; D.A. Aliev leg.; LE: 137 (3). • Lenkoran District, swamps soil, grown in culture; 28 February 1962; L.V. Bayramova leg.; pressed 10 September 1962; L.K. Krassavina; LE: 161 (1).

Published records: AZ [26,34,35,37].

Habitat: Rice fields, small inland and coastal water bodies, also recovered from soil diaspore bank.

*Chara canescens* Desv. et Lois. in Lois. (Figure 1A)

=*C. crinita* Wallr.

Material examined: Armenia • Lake Sevan, Gyuney [Areguni]; 14 May 1938; G.M. Fridman leg.; LE: 28 (1). • Lake Sevan, Elenovskaya Bay, near island, at sand split; 14 May 1938; G.M. Fridman leg.; LE: 28 (2). • Vedi Region, Arazdayan [Yeraskh], ditch; 18 July 1954; Academia Scientiarum Arm. SSR. Hortus Botanicus Erevanensis; LE. • Azerbaijan • Bolshoy Bay [Gizylagach Bay], station No. 10, bay mouth, near the end of the Kurinskaya Split; 3 August 1947; K.[A.] Dunina leg.; LE: 82 (13). • Bolshoy Bay [Gizylagach Bay], the western shore of the bay, at the profile “Gavrilovskie Koshi”—“Arakelovskiy Bank”; 4 August 1947; K.[A.] Dunina leg.; LE: 82 (18). • Maliy Bay [Small Gizylagach Bay], near the eastern extremity of “Neizvestniy” Island, near Semchenkov Kultuk; 8 August 1947; K.[A.] Dunina leg.; LE: 82 (11). • Ghizil-Agaj State Reserve, Gizylagach Bay; 14 October 1949; V.V. Veber leg.; LE: 87 (2). • Absheron Peninsula, station Plyazh [beach, near the village of Buzovna], wetlands; 12 May 1958; D.A. Aliev leg.; LE: 143 (3). • Russia • Krasnodar Territory, Anapa District, the settlement of Maliy Utrish, a small shallow brackish lake near the seashore; 44.7090° N; 37.4558° E; 30 May 2021; N.S. Gamova leg.; LE: A0001522.

Published records: Armenia [22,31,42,43], Azerbaijan [26], Georgia [38,44].

Habitat: Sea bays, brackish coastal waters, freshwater lakes, and channels.

*Chara connivens* Salzm. ex A. Braun (Figure 1B)

Material examined: Georgia • Tiflis Guberniya, Tiflis Uezd, Saguramo Ridge, a saline [temporary?] lake near the monastery of Saint Crux [Lake Jvari-Tbo near Jvari]; 11 July 1910; V. Kozlovskiy leg.; Ex herbario Horti Botanici Tiflisiensis; LE.

Published records: Georgia [34,35,37,38], Russia [14].

Habitat: Small inland lakes; large freshwater mountain and coastal water reservoirs.

*Chara contraria* A. Braun ex Kütz. var. *contraria* (Figure 1B)

Material examined: See Appendix A.

Published records: Armenia [27,28,29,31,45,46,47,48,49], Azerbaijan [26,30,50], Georgia [36,37,38,51], Russia [14,52].

Habitat: Small freshwater lakes, including limnocrenic and mountain ones; small water bodies and ponds, including low-lying areas surrounded by a rampart where water accumulates during the spring rains or partly accumulates from rivers, and the water is used for rice fields (istil is a local name for this type of water body).

Notes: Some specimens, i.e., from Lake Kustba in Georgia, and Lakes Maralgol, Zeligol, Shamlygyol in Azerbaijan, are robust coarse plants similar to *Chara papillosa* Kütz. sensu Groves et Groves (=*C. intermedia* A. Braun) [53], making their delineation tricky. They have very short, solitary spine-cells. However, their re-identification as *C. papillosa* cannot be ruled out, if more plants from these sites could be checked in the future. Moreover, both *C. contraria* and *C. papillosa* can grow side-by-side.

*Chara contraria* var. *hispidula* A. Braun (Figure 1B)

Material examined: Azerbaijan • Khanlar District [Goygol District, Goygol National Park], M.K. Dzhiali-gel’ [Lake Dzhali-gyol, Dzhaligol]; 14 July 1970; F.A. Babaev leg.; LE. • Khanlar District [Goygol District, Goygol National Park], M.K. Gek-gel’ [Lake Göygöl]; 14 July 1970; F.A. Babaev leg.; LE.

Habitat: Freshwater mountain lakes.

Floristic novelty: New variety recorded for the Caucasus.

*Chara denudata* A. Braun (Figure 1B)

Material examined: See [54].

Published records: Azerbaijan [54].

Habitat: Probably a river or small water body associated with a river.

*Chara globata* Migula (Figure 2A)

Material examined: Russia • Prov. Mari-Nigri.—500 lih ** P *** [illegible letters] Eusini pr. Sladky Liman [vicinity of Abrau-Dyurso, Lake Sladkiy Liman]; 22 July 1925; N. Wwedensky leg., No. 1102 Charae Caucasicae; LE. • Republic of Dagestan, Samur Forest, a freshwater water body, a source of water for sturgeon farming; pulled ashore after clearance of water body; 41.8663° N; 48.5452° E; 3 October 2022; O.N. Boldina leg.; LE.

Published records: Armenia [55], Russia [14].

Habitat: Permanent freshwater large and small lakes; small freshwater and brackish coastal lakes and water bodies.

*Chara globularis* Thuill. (Figure 2A)

=*C. fragilis* Desv.

Material examined: Georgia • [Abkhasia], Gagra, the road to Cape Pitsunda, a pond among the fields to the right of the road before reaching Bzyb [Bzipi], in very shallow water, on clay; 23 May 1940; V.L. Komarov leg.; LE: A0001315, LE: A0001316. • Russia • Republic of Adygea, Caucasus Nature Reserve, West Caucasus, drainage basin of Belaya River, Guzeripl Mountain, lake ~20 × 30 m in suffusion funnel; 43.9813° N; 39.9619° E; alt: 1960 m a.s.l.; 9 September 2021; T.V. Akatova leg.; LE. • Republic of Dagestan, Samur Forest, a freshwater water body, a source of water for sturgeon farming; pulled ashore after clearance of water body; 41.8663° N; 48.5452° E; 3 October 2022; O.N. Boldina leg.; LE; sterile plants.

Published records: Armenia [31], Azerbaijan [26,56,57], Georgia [36,37,58].

Habitat: Freshwater lakes of different types, streams, ponds, and istiles (see explanation of this local name in the note for *C. contraria* var. *contraria*).

*Chara gymnophylla* A. Braun (Figure 2B)

= *C. vulgaris* ssp. *gymnophylla* (A. Braun) A. Braun

Material examined: See Appendix A. Once was reported as *C. foetida* A. Braun [59].

Published records: Armenia [31,45,49]; Azerbaijan [60,61,62,63]; Georgia [34,35,37,38,64,65,66,67,68] (as *C. vulgaris* ssp. *gymnophylla*); Russia [14,69] (probably incl. record of *C. foetida*? A. Braun).

Habitat: Freshwater small water bodies, wetlands, ponds, springs, streams, rivers, and water bodies associated with them. The records from high mountain lakes need confirmation.

*Chara hispida* L. (Figure 2A)

Material examined: Not available.

Published records: Azerbaijan [37,70].

Habitat: Brackish swamp.

Note: It would be desirable to check this old record in Azerbaijan, based on the specimen identified by F. Kützing [70], considering that species concepts change over time [39]. However, its voucher has not been located yet.

*Chara neglecta* Hollerbach (Figure 2B)

Material examined: Azerbaijan • Bolshoy Bay [Gizylagach Bay], the eastern shore of the bay, near “Zeynal-Kurasy”; 29 July 1946; K.[A.] Dunina leg.; LE: 82 (6). • Bolshoy Bay [Gizylagach Bay], the western shore of the bay, at the profile “Gavrilovskie Koshi”—“Arakelovskiy Bank”, near “Arakelovskiy Bank”; 25 October 1946; K.[A.] Dunina leg.; LE: 82 (2). • Maliy Bay [Small Gizylagach Bay], station No. 3, in the middle of the profile collective farm “Shirin-Kuly” (“Port Ilyicha”)—“Sarinskaya M.R.S.”; 3 November 1946; K.[A.] Dunina leg.; LE: 82 (5). • Bolshoy Bay [Gizylagach Bay], the eastern shore of the bay, station No. 12, at the profile “Gavrilovskie Koshi”—“Arakelovskiy Bank”, closer to “Gavrilovskie Koshi”; 3 August 1947; K.[A.] Dunina leg.; LE: 82 (14). • Bolshoy Bay [Gizylagach Bay], the western shore of the bay, at the profile “Zeynal-Kurasy”—“Gavrilovskie Koshi”, near “Zeynal-Kurasy”; 4 August 1947; K.[A.] Dunina leg.; LE: 82 (8). • Bolshoy Bay [Gizylagach Bay], the eastern shore of the bay, at the profile “Zeynal-Kurasy”—“Gavrilovskie Koshi”, closer to “Gavrilovskie Koshi”; 4 August 1947; K.[A.] Dunina leg.; LE: 82 (7). • Bolshoy Bay [Gizylagach Bay], the western shore of the bay, at the profile “Gavrilovskie Koshi”—“Arakelovskiy Bank”; 4 August 1947; K.[A.] Dunina leg.; LE: 82 (18). • Bolshoy Bay [Gizylagach Bay], the eastern shore of the bay, at the profile “Zeynal-Kurasy”—“Gavrilovskie Koshi”; 4 August 1947; K.[A.] Dunina leg.; LE: 82 (9). • Maliy Bay [Small Gizylagach Bay], near the eastern extremity of “Neizvestniy” Island, near Semchenkov Kultuk; 8 August 1947; K.[A.] Dunina leg.; LE: 82 (11). • Bolshoy Bay [Gizylagach Bay], station No. 6, opposite “Ivanovskiy” Island; 30 October 1947; K.[A.] Dunina leg.; LE: 82 (12). • Bolshoy Bay [Gizylagach Bay], the western shore of the bay, at the profile “Gavrilovskie Koshi”—“Arakelovskiy Bank”, near “Arakelovskiy Bank”; 3 November 1947; K.[A.] Dunina leg.; LE: 82 (17).

Published records: Azerbaijan [71], Russia [14].

Habitat: Sea bays and brackish coastal water bodies.

*Chara papillosa* Kütz. (Figure 2B)

=*C. intermedia* A. Braun

=*C. aculeolata* auct. non Kütz.

Material examined: Armenia • Lake Sevan [Areguni coast]; 19 September 1938; G.M. Fridman leg.; LE: 23 (2), LE: 23 (3), LE: 23 (4), LE: 23 (5). • Lake Sevan, near Naroshen [Norashen]; 17 September 1939; G.M. Fridman leg.; LE: 28 (2). • Azerbaijan • In regione subalpina montis Kaepes-Dagh [Kepez Daghi, Mount Kapaz], provinciae Karabach [Goygol District, Goygol National Park]. In lacu Schaloch-ghöll [Lake Shamlygyol], 8–9000′ [feet, ~2438–2743 m a.s.l.]; 12 July 1844; Dr. [F.A.R.] Kolenati leg., Fl. Transcauc. No. 1970; LE. • Khanlar District [Goygol District, Goygol National Park], M.K. Ordekgel [Lake Ordekgol]; 12 July 1970; F.A. Babaev leg.; LE. • Kelbalzharsky District [Kalbajar District], M.K. Ganlygel [probably Kanligel—Lake Gortagarak]; 13 July 1970; F.A. Babaev leg.; LE. • Georgia • Vicinity of Tbilisi, Lake Lisis-Tba [Lake Lisi], northern part; 13 June 1939; T.E. Dzhibladze leg.; LE: 41 (1). • Vicinity of Tbilisi, Lake Lisis-Tba [Lake Lisi], northern part; 21 June 1939; T.E. Dzhibladze leg.; LE: 41 (3). • Russia • Kabardino-Balkarian Republic, Tersk Region, Nalchik, vic[inity], alp[ine] Lake Tzirik [Lake Cerik-Köl, Lake Nizhnee Goluboe]; 26 Jule 1893; W. Lipsky leg., Flora Caucasica; LE. • Balkaria, lake at 3000′ [feet, ~914 m a.s.l.], 26 Jule 1893; I. Akinfiew leg., Herbarium caucasicum, 1882–1897; LE. • Lake Cerik-Köl, station VIII, near small river source; September 1926; I.G. Kusnetzov leg., Comite Geologique de Russie; LE.

Published records: Armenia [31,43,47,48,72,73,74]; Azerbaijan [34] (as *C. intermedia* A. Braun ssp. *aculeolata* Mig. f. *pumila* Mig.), [37]; Georgia [38,51]; Russia [75].

Habitat: Freshwater large and small lakes with high water transparency.

Notes: The records of *C. subspinosa* Rupr. as *C. rudis* A. Braun for Georgia and the Caucasus are based on the specimens of *C. papillosa* from Lake Lisis-Tba/Lake Lisi [51,76]. Their re-identification allows for the exclusion of *C. subspinosa* from the Caucasian flora of charophytes. The record from Gelendzhik Bay [75] needs confirmation. An old specimen from Lake Shamlygyol was reported by Ruprecht [77] and Petunnikow [34] as *C. vulgaris* Smith. A peculiar, slender, elongated morphotype with short branchlets was collected in the limnocrene Lake Cerik-Köl.

*Chara squamosa* Desf. (Figure 3A)

Material examined: Georgia • Imeretia, prope Utsera fl. Rion[i]; July 1877; A.H. Brotherus leg.; H 50030099.

Published records: Russia [78].

Habitat: In streams and rivers.

Floristic novelty: New species record for the South Caucasus.

Notes: An old specimen from the vicinity of Utsera was reported by Braun and Nordstedt [68] as *C. vulgaris* ssp. *gymnophylla*. The distribution area of this species is insufficiently known because of the recently re-evaluated species concept [39]. Considering its association with hilly and mountainous environments in the Balkans and across the Mediterranean [79,80], it should be expected in more sites within the Caucasus. Some reported sites of *C. gymnophylla* could belong to this species.

*Chara strigosa* A. Braun (Figure 2A)

Material examined: Not available.

Published records: Azerbaijan [34,37].

Habitat: Mountain lakes with high water transparency.

Note: This record, isolated from the species distribution range [81], was probably based on another species. Examining its voucher would clarify this.

*Chara tomentosa* L. (Figure 1B)

Material examined: Not available.

Published records: Armenia [72], Russia [82].

Habitat: Mountain lakes, and ponds with high water transparency.

*Chara uzbekistanica* Hollerbach (Figure 3A)

Material examined: Armenia • Lake Sevan, Gyuney [Areguni] coast; 14 May 1938; G.M. Fridman leg.; LE: 28 (1). • Lake Sevan [Areguni coast]; 19 September 1938; G.M. Fridman leg.; LE: 23 (4). • Lake Sevan [Areguni coast]; 19 September 1938; G.M. Fridman leg.; LE: 23 (3), LE: 23 (2 bis), LE: 23 (1). • Lake Sevan, near Noraduz [Noratus]; 26 November 1938; G.M. Fridman leg.; LE: 28 (6). • Lake Sevan, near Noraduz [Noratus]; 17 September 1939; G.M. Fridman leg.; LE: 28 (6). • Lake Sevan, near Babadzhan [Babajan, Kyzylkend, Tsapatagh]; 20 September 1939; G.M. Fridman leg.; LE: 28 (5), LE: 28 (7). • Lake Sevan, near Zagalu [Tzovak, Tsovak]; 22 September 1939; G.M. Fridman leg.; LE: 28 (8), LE: 28 (3). • Lake Sevan, north of Lchashenskaya Bay; 7 October 2006; A.A. Bobrov leg.; IBIW 54128.

Habitat: Large freshwater lakes with high water transparency and probably associated water bodies.

Floristic novelty: New species record for the Caucasus.

Note: All studied specimens from Lake Sevan referred earlier to *C. globularis* were found to be misidentifications of *C. uzbekistanica*. Based on studied specimens and images available at iNaturalist.org [83], we expect that at least some of published records of *C. globularis* from Lake Sevan [20,22,31,43,47,72,73,84] belong to *C. uzbekistanica*. This suggestion was tentatively accepted for map preparation. However, it could be tested if new collections will be available for study.

*Chara virgata* Kütz. (Figure 3A)

Material examined: Azerbaijan • Masally District, the village Gegachol [Gegechol, Giga-Chel], in istil [see explanation of this local name in the note for *C. contraria* var. *contraria*]; 2 June 1958; D.A. Aliev leg.; LE: 144 (2).

Habitat: Artificial pond with variable water level.

Floristic novelty: New species record for the Caucasus.

*Chara vulgaris* L. f. *vulgaris* (Figure 3A)

=*C. foetida* A. Braun

Material examined: See Appendix A.

Published records, partly as *C. foetida*: Armenia [27,28,29,31,45,46,47,48,74,85], Azerbaijan [26,30,50,70,86,87,88,89], Georgia [34,35,36,37,38,68,77,90,91,92,93], Russia [14,32,52,94,95,96,97,98,99].

Habitat: A wide spectrum of freshwater habitats, from small temporary water bodies to large lakes, mainly small water bodies and rivers.

Note: Some records of *C. contraria* from Azerbaijan reported by Vilhelm [36] are based on specimens of *C. vulgaris* collected by Alexeenko. The records from the Sudzhuk Lagoon (since [94]) could be based on the plants of *C. baltica*, but no vouchers for the survey by Arnoldi [94] were located.

*Chara vulgaris* f. *longibracteata* (Kütz.) H. Groves (Figure 3A)

Material examined: See Appendix A.

Published records: Russia [14,100] (as *C. vulgaris*).

Habitat: The same as in the case of f. *vulgaris*, excluding lakes (see above).

*Lamprothamnium papulosum* (Wallr.) J. Groves (Figure 3B)

= *Lamprothamnus alopecuroides* Delile ex. A. Braun et Nordstedt

= *Lamprothamnus alopecuroides* var. *tenuispina* Arnoldi

Material examined: Azerbaijan • Bolshoy Bay [Gizylagach Bay], the eastern shore of the bay, at the profile “Gavrilovskie Koshi”—“Arakelovskiy Bank”, closer to “Gavrilovskie Koshi”; 24 October 1946; K.[A.] Dunina leg.; LE: 82 (1). • Bolshoy Bay [Gizylagach Bay], the western shore of the bay, station No. 2, at the profile “Zeynal-Kurasy”—“Gavrilovskie Koshi”, closer to “Zeynal-Kurasy”; 26 October 1946; K.[A.] Dunina leg.; LE: 82(3). • Maliy Bay [Small Gizylagach Bay], station No. 3, in the middle of the profile collective farm “Shirin-Kuly” (“Port Ilyicha”)—“Sarinskaya M.R.S.”, near the shore of “Port Ilyicha”; 3 November 1946; K.[A.] Dunina leg.; LE: 82 (4). • Maliy Bay [Small Gizylagach Bay], station No. 3, in the middle of the profile collective farm “Shirin-Kuly” (“Port Ilyicha”)—“Sarinskaya M.R.S.”; 3 November 1946; K.[A.] Dunina leg.; LE: 82 (5). • Bolshoy Bay [Gizylagach Bay], the western shore of the bay, station No. 13, at the profile “Gavrilovskie Koshi”—“Arakelovskiy Bank”, closer to “Arakelovskiy Bank”; 29 July 1947; K.[A.] Dunina leg.; LE: 82 (15). • Bolshoy Bay [Gizylagach Bay], the eastern shore of the bay, station No. 12, west coast, at the profile “Gavrilovskie Koshi”—“Arakelovskiy Bank”; 3 August 1947; K.[A.] Dunina leg.; LE: 82 (14). • Bolshoy Bay [Gizylagach Bay], station No. 10, bay mouth, near the end of the Kurinskaya Split; 3 August 1947; K.[A.] Dunina leg.; LE: 82 (13). • Maliy Bay [Small Gizylagach Bay], near the eastern extremity of “Neizvestniy” Island, near Semchenkov Kultuk; 8 August 1947; K.[A.] Dunina leg.; LE: 82 (11). • Bolshoy Bay [Gizylagach Bay], the western shore of the bay, station No. 13, at the profile “Gavrilovskie Koshi”—“Arakelovskiy Bank”, closer to “Arakelovskiy Bank”; 3 October 1947; K.[A.] Dunina leg.; LE: 82 (16). • Bolshoy Bay [Gizylagach Bay], station No. 6, opposite “Ivanovskiy” Island; 30 October 1947; K.[A.] Dunina leg.; LE: 82(12). • Bolshoy Bay [Gizylagach Bay], station No. 5, near “Kabanya sand split”; 30 November 1947; K.[A.] Dunina leg.; LE: 82 (10). • Russia • Krasnodar Territory, Novorossiysk, Sudzhuk Lagoon; 20 July 1925; A.D. Zinova, V.S. Stefanov leg.; LE. • Krasnodar Territory, Novorossiysk, Sudzhuk Lagoon; 21 September 1953; A.D. Zinova, V.S. Stefanov leg.; LE.

Published records: Russia [18,25,32,33,37,94,95,96,97,98,101,102].

Habitat: Brackish seashore lagoons and sea bays.

Note: The last record from the Sudzhuk Lagoon, supported by the available specimens, dates back to 1953. Therefore, we cannot exclude the possibility that at least some of the subsequent records from this site may be misidentifications of *Chara*, considering the severe changes experienced by this water body over time [32,33,95,97,101,103] and the narrow ecological niche of *L. papulosum*, which led to the decline of this species across its distribution range [39].

*Nitella capillaris* (Krock.) J. Groves et Bull.-Webst. (Figure 3B)

= *N. capitata* (Nees) C. Agardh

Material examined: Azerbaijan • Lenkoran D[istrict], rice fields in the vicinity of the village of Kumbashi [Qumbaşı], in a water body near the dam of istil [see explanation of this local name in the note for *C. contraria* var. *contraria*]; 12 February 1959; T. Kutova leg.; LE: 141 (4).

Published records: Georgia [37,58].

Habitat: Rice fields, oxbow lakes.

*Nitella flexilis* (L.) C. Agardh (Figure 3B)

Material examined: Russia • [Kabardino-Balkarian Republic], Teberda Reserve, Lake Karakel’ [Lake Kara-Kol]; March 1939; V.L. Komarov leg.; LE.

Habitat: Small mountain lake.

*Nitella mucronata* (A. Braun) Miq. (Figure 3B)

Material examined: Georgia • Sukhumi Okrug [Gulripshi municipality], Babuscheri [Babushara]; 18 August 1899; Voronov [Woronow] leg., Hortus Botanicus Tiflisiensis; Herbarium cryptogamicum; LE. • Russia • Kuban Region [Krasnodar Territory], [stanitsa of] Krymskaya [the town of Krym]; 8 June 1891; V. Lipsky leg.; LE: A0001306.

Habitat: Not indicated on labels.

Note: The record of *N. opaca* for Georgia [34] is based on the studied specimen of *N. mucronata*.

*Nitella opaca* (C. Agardh ex Bruzelius) C. Agardh

Material examined: Not available.

Published records: Azerbaijan [36], Georgia [34,38].

Habitat: Lakes.

Note: See note for *N. mucronata* about the record for Georgia. The record for Azerbaijan [36] is based on sterile *Nitella*, either *N. flexilis* or *N. opaca*. Therefore, the species should be excluded from the list of charophytes in Azerbaijan. This species is known in Georgia only from online resources [83,104].

*Nitellopsis obtusa* (Desvaux) J. Groves

Material examined: Not available.

Habitat: Lakes.

Note: This species is known in Georgia only from online resources [83,104].

*Sphaerochara prolifera* (Ziz ex A. Braun) Soulié-Märsche (Figure 3B)

=*Tolypella prolifera* (Ziz ex A. Braun) Leonh.

Material examined: Not available.

Published records: Georgia [105].

Habitat: Brackish water bodies.

*Tolypella glomerata* (Desv. in Loisel.) Leonh. (Figure 3B)

Material examined: Azerbaijan • Lenkoran District, Malyy Kyzyl-Agach Bay with fresh water, lagoons inside reeds and cattails; 6 February 1959; T.[N.] Kutova leg.; LE: 141 (1). • Lenkoran District, Island Sara, puddle-like freshwater bodies on the shore of the Bolshoy Kyzyl-Agach Bay separated from the bay water by a ridge of shell rock and a strip of rushes; 7 February 1959; T.[N.] Kutova leg.; LE: 141 (2), LE: 141 (3). • Apsheron Peninsula, the railway station of Zagul’ba, small water body; 9 May 1958; D.A. Aliev leg.; LE: 137 (1); LE: 143 (1).

Published records: Azerbaijan [26].

Habitat: Small coastal water bodies.

*Tolypella nidifica* (O.F.Müll.) A. Braun (Figure 3B)

Material examined: Russia • Krasnodar Territory, Black Sea, Caucasian coast, Anapa Bay; 1957; K.M. Petrov leg.; LE.

Published records: Russia [14,106,107].

Habitat: Small brackish coastal water bodies, sea bays.

**Figure 5:**
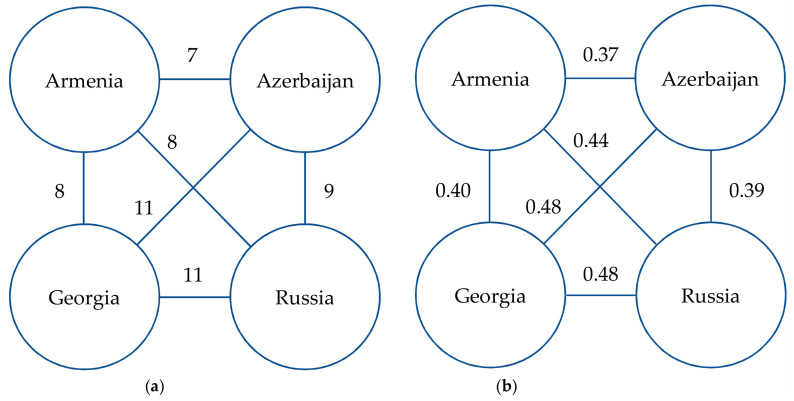
Similarity of charophyte floras of Caucasus countries: (**a**) number of species common for the compared regions; (**b**) values of Jaccard index.

The records based on specimens collected before 1979 predominate in the dataset of Caucasian charophytes (Figure 4). Based on all the available references and studied specimens, the Caucasian charophyte flora counts 27 species from six genera (Table 1), including 10 species in Armenia, 16 species in Azerbaijan, 18 species in Georgia, and 16 species in Russia. Only six species are known in all Caucasian countries. The low similarity of the charophyte floras among the Caucasian countries (Figure 5) could rather reflect different degrees of knowledge.

## 3. Discussion

### 3.1. Species Composition

The high species richness of the genus *Chara* L., the much less diverse genus *Nitella* Agardh, and a few species of *Tolypella* (A. Braun) A. Braun and *Sphaerochara* Mädler in the Caucasian charophyte flora are typical traits of Palearctic charophyte floras, except East Asian ones [5,7,8,9,11,12,13,14,39,108,109,110,111,112]. However, the checking of vouchers in collections not covered by this research would be desirable, because some misidentifications could be expected from the perspective of the distribution range and ecology of a few of the species recorded in the Caucasus.

The species richness of the Caucasian charophytes seems to remain incompletely known. In particular, at least one dioecious species of *Tolypella* and some species of *Chara* from the subsection *Hartmania* R.D. Wood could be expected in the Caucasian flora, considering their distribution ranges [39,113] (Table 2). In comparison, 37 species of charophytes from seven genera are known in Montenegro [80], having a notable combination of diverse charophyte habitats from the Adriatic coast to the Dinaric Alps in a smaller area than the Caucasus, which is also rich in different potential charophyte habitats.

The plains north of the Caucasus harbor at least 29 species of charophytes according to current taxonomy (Table 2), excluding an erroneous record of *Nitella tenuissima* (Desv.) Kütz. [114]. This number is similar to the known species richness of the Caucasian charophytes. The flora of the southeastern part of the East European Plain differs by the absence of records of *Chara denudata*, *C. gymnophylla*, *C. hispida*, *C. squamosa*, *Nitella capillaris*, *N. flexilis*, and *Tolypella nidifica*. From the perspective of species ecology, it could be hypothesized that the Caucasus harbors the northern borders of the distribution areas of *C. denudata*, *C. gymnophylla*, and *C. squamosa*. The Caucasian records for *C. hispida*, *C. strigosa*, *N. capillaris*, and *N. flexilis* could probably be recognized as species exclaves with wide disjunctions to the main parts of these species’ distribution areas, situated more westward and northward of the Caucasus. Reliable records for *T. nidifica* in the south of Eastern Europe and the Caucasus are only associated with the brackish waters of seashores.

In contrast, the area north of the Caucasus has populations of *Chara altaica* A. Braun in A. Braun et Nordst., *C. aspera* Willd., *C. baueri* A. Braun, *C. kirghisorum* Lessing, *C. oedophylla* Feldm.-Maz., *C. tenuispina* A. Braun, *Lychnothamnus barbatus* (Meyen) Leonh., *Nitella gracilis* (Smith) C. Agardh, *N. hyalina* (DC.) C. Agardh, *Sphaerochara intricata* (Trentep. ex Roth) Feist-Castel & N. Grambast, and *Tolypella mongolica* R.E. Romanov, V.S. Vishnyakov, V.Yu. Nikulin et A.A. Gontcharov (Table 2), unknown in the Caucasus. Some of them, but not all, could be expected in the studied area. It could be concluded that the charophyte floras of these large regions are not identical, although most of the species are known in both of them. The combination of species distinguishing the Caucasian charophyte flora from the southeastern part of the East European Plain is represented by species having mainly Mediterranean (*C. squamosa*), Mediterranean–West Asian (*C. gymnophylla*), European (*C. hispida*, *N. capillaris*, and *T. nidifica*), Holarctic (*N. flexilis*), Palearctic (*C. papillosa*, *C. strigosa*), and Afro-West Asian (*C. denudata*) distribution ranges, and not by endemic or subendemic species.

**Table 2:** Species lists of charophytes of the Caucasus, the southeastern part of the East European Plain, Türkiye [Turkey], Iran, and their general distribution.

Species	Caucasus	SE EEP	Türkiye	Iran	General Distribution
*Chara aculeolata* Kütz. in Rchb.	–	–	–	+	Europe; North and Southeast Africa: Mozambique; West and Central Asia
*Chara altaica* A. Braun in A. Braun et Nordst.	–	+	–	–	Eastern Europe, South Ural, South Siberia, Central and East Asia
*Chara aspera* Willd.	–	+	+	–	Holarctic
*Chara baltica* (Hartm.) Bruz.	+	+	+	+	North, Central, and East Europe; North Africa; West and Central Asia; Greenland
*Chara baueri* A. Braun	–	+	–	–	Europe; Central Asia: Kazakhstan
*Chara braunii* C.C. Gmelin	+	+	–	+	Cosmopolitan
*Chara canescens* Desv. et Lois. in Lois.	+	+	+	+	Holarctic, recovered from diaspore bank in Australia
*Chara connivens* Salzm. ex A. Braun	+	+	+	+	Europe, North Africa, Asia
*Chara contraria* A. Braun ex Kütz.	+	+	+	+	Subcosmopolitan
*Chara corfuensis* J. Groves	–	–	+	–	Mediterranean: Europe; North Africa and West Asia; East Europe
*Chara denudata* A. Braun	+	–	–	–	North and South Africa, West Asia
*Chara fibrosa* C. Agardh ex Bruz. s.l.	–	–	–	+	Pantropical, Europe, West and Central Asia
*Chara grovesii* B.P. Pal	–	–	–	+	West and Southeast Asia: Iran and Myanmar
*Chara globata* Migula	+	+	–	+	Southeast and East Europe; North Africa; West, Central, North, and East Asia
*Chara globularis* Thuill.	+	+	+	–	Cosmopolitan
*Chara gymnophylla* A. Braun	+	–	+	+	South Europe, Mediterranean, West and East Asia, South Africa, South America
*Chara hispida* L.	+	–	+	–	Europe, North Africa, West Asia
*Chara imperfecta* A. Braun in Durieu	–	–	+	–	West and South Europe, North Africa, West Asia
*Chara kieneri* Daily	–	–	+	–	West Asia: Türkiye; North America
*Chara kirghisorum* Lessing	–	+	–	+	East Europe; North, West, and Central Asia
*Chara kohrangiana* A. Ahmadi, M. Sheidai, H. Riachi, J.C. van Raam	–	–	–	+	West Asia: Iran
*Chara neglecta* Hollerbach	+	+	+	–	East Europe; West, North, and Central Asia
*Chara oedophylla* Feldm.-Maz.	–	+	–	–	Mediterranean, East Europe
*Chara papillosa* Kütz.	+	–	+	–	Palearctic
*Chara socotrensioides* (R.D. Wood) R.D. Wood	–	–	–	+	West and South Asia: Iran and Myanmar
*Chara squamosa* Desf.	+	–	+	–	South Europe, North Africa, West Asia
*Chara strigosa* A. Braun	+	–	–	–	Palearctic
*Chara tenuispina* A. Braun	–	+	+	–	Eurasia
*Chara tomentosa* L.	+	+	+	+	Palearctic
*Chara uzbekistanica* Hollerbach	+	+	–	+	East Europe, West and Central Asia
*Chara virgata* Kütz.	+	+	+	–	Cosmopolitan
*Chara vulgaris* L.	+	+	+	+	Cosmopolitan
*Chara zeylanica* Willd. s.l.	–	–	+	–	Pantropical; South Europe: Sardinia; West Asia
*Lamprothamnium papulosum* (Wallr.) J. Groves	+	+	–	+	Eurasia, North Africa
*Lychnothamnus barbatus* (Meyen) Leonh.	–	+	–	–	Eurasia, North America, Australia
*Nitella capillaris* (Krock.) J. Groves et Bull.-Webst.	+	–	–	–	Europe, West Asia
*Nitella flexilis* (L.) C. Agardh	+	–	–	–	Subcosmopolitan
*Nitella gracilis* (Smith) C. Agardh	–	+	+	–	Cosmopolitan
*Nitella hyalina* (DC.) C. Agardh	–	+	–	+	Cosmopolitan
*Nitella mucronata* (A. Braun) Miq.	+	+	+	–	Cosmopolitan
*Nitella opaca* (C. Agardh ex Bruzelius) C. Agardh	+	+	+	–	Cosmopolitan
*Nitellopsis obtusa* (Desvaux) J. Groves	+	+	+	–	Eurasia, North Africa, invasive in North America
*Sphaerochara intricata* (Trentep. ex Roth) Feist-Castel & N. Grambast	–	+	–	–	Holarctic
*Sphaerochara prolifera* (Ziz ex A. Braun) Soulié-Märsche	+	+	–	–	Holarctic
*Tolypella glomerata* (Desv. in Loisel.) Leonh.	+	+	+	+	Subcosmopolitan
*Tolypella hispanica* Norsdt. ex Allen	–	–	–	+	Mediterranean; West Asia: Iran
*Tolypella iranica* R.E. Romanov, V.Yu. Nikulin et A.A. Gontcharov	–	–	–	+	West Asia: Iran
*Tolypella mongolica* R.E. Romanov, V.S. Vishnyakov, V.Yu. Nikulin et A.A. Gontcharov	–	+	–	–	East Europe, South Siberia, Central Asia
*Tolypella nidifica* (O.F.Müll.) A. Braun	+	–	–	–	Europe, West Asia
Species number	27	29	24	21	

Note: A comprehensive bibliography is impossible to report within the scope of this article, so the cited references include all the known species from the territories. References: Caucasus (this study). • SE EEP: the southeastern part of the East European Plain (plain areas of Krasnodar and Stavropol territories; Astrakhan and Volgograd regions; and republics of Kalmykia, Adygeya, and Dagestan) [14,39,115,116,117]. • Türkiye: [118,119,120,121,122,123,124]. The record of *C. polyacantha* A. Braun ex Braun, Rabenhorst et Stizenberger [123] belongs to *C. corfuensis* according to the published images and description. • Iran: [113,122,125,126,127,128,129]. The species’ general distributions are tentatively outlined based on [54,55,110,113,124,127,128,129,130,131] (and references within).

**Figure 6:**
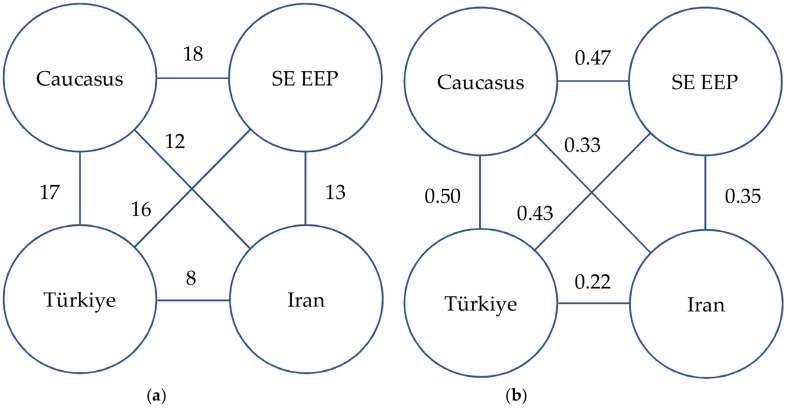
The similarity of the charophyte floras of the Caucasus and neighboring regions: (**a**) number of species common to all regions; (**b**) values of the Jaccard index. SE EEP—the southeastern part of the East European Plain.

Few records are available from the Caucasus in Türkiye—*Nitella opaca* [36] (LE, checked), and Iran—*Chara gymnophylla*, *C. vulgaris*, and *Tolypella hispanica* [70,122,128]. However, the Turkish charophytes are represented by 24 species, and the Iranian charophyte flora consists of 21 species (Table 2). The charophyte floras of Türkiye and Iran are somewhat overlapping, but neither one of them is identical to the Caucasian one. They are slightly smaller in contrast to the Caucasian flora (Table 2), but the Iranian flora has two recently described species, *C. kohrangiana* and *T. iranica*. They can be tentatively recognized as national endemics. Each compared region has a few, 4–7, species known only to it, resulting in the dissimilarity of the compared floras and providing evidence for the moderate uniqueness of the species combinations in each region. Only seven species are common among the charophyte floras of the Caucasus, the southeastern part of the East European Plain, Türkiye, and Iran. The Caucasus has a middle level of similarity with the charophyte flora of the southeastern part of the East European Plain and Türkiye (Figure 6). Therefore, the Caucasian charophyte flora could be described as not unique from a large-scale perspective, i.e., within Eurasia, considering the species distribution ranges. However, the association of species makes the region special at the scale of West Asia comparing to large neighboring regions. This moderate distinctiveness of the Caucasian charophyte flora, lacking endemic species, contrasts with the more distinctive Caucasian angiosperm flora, which includes many endemics [132,133,134,135,136]. Such low distinctiveness is found in charophyte floras of Tajikistan [137], Dagestan [14], and the Balkans [138,139], considering the current nomenclature [39].

### 3.2. Distribution and Species Frequency

The predominance of records based on specimens collected before 1979 is apparent in the dataset for Caucasian charophytes (Figure 4B). However, these records can serve as an important baseline for the estimation of trends in occurrence frequency and distribution, as well as for tracking changes in particular water bodies if new surveys are implemented. The rarity of specialist species with narrow ecological niches having a restricted distribution in the Caucasus is evident from the data available (Figure 1, Figure 2 and Figure 3). It appears to reflect both the well-pronounced spatial environmental heterogeneity of the area studied and poor knowledge of the charophytes in many regions.

It is unclear how frequently associations of more than three species occur at the same sites. *Chara contraria*, *C. gymnophylla*, and *C. vulgaris* are the most widely distributed species in the Caucasus (Figure 1, Figure 2 and Figure 3). Most of the records and sites of Caucasian charophytes belong to them. This allows for their recognition as generalist species in the region studied, suitable for many temperate areas, except *C. gymnophylla*. It is mostly associated with flowing water, water bodies like river and stream small branches, and water seepages near rivers and streams. The records for it from deep lakes need confirmation. The frequent occurrence of *C. gymnophylla* is typical in the Mediterranean and West Asia [39,124,128,140] (and references within). *Chara vulgaris* is the most common species in all the Caucasian states, which appears to be a common pattern with other areas of West and Central Asia, the semiarid and arid regions of inland Eurasia, and the Mediterranean [39,111,124,137,140]. The rare occurrence of *C. globularis* is evident in comparison to, e.g., the forest and forest–steppe biomes of Europe [39]. This species has a maximal number of records in Armenia (Figure 2A), but none of them can be confirmed because no vouchers are traceable at the moment. *Chara squamosa* is only known from a few sites, but it should be expected in all Caucasian countries.

The habitats of the Caucasian charophytes generally fit the environmental patterns known for these species before [39] (and references within). Diverse aquatic habitats [31,141,142,143,144] that have emerged from orographic and geological complexity, and variability in elevation, exposition, precipitation, climate traits, etc., both at a large and small scale [145], have resulted in combinations of charophyte species with different distribution ranges, life strategies, and habitat requirements, similar to the aquatic and wetland angiosperms of the Caucasus [31,143,146]. Deep and shallow transparent lakes, ponds and small water bodies, sea bays and seashore water bodies, springs, streams, small rivers, and associated water bodies are important habitats of the charophytes in the Caucasus. Future charophyte surveys should be focused on them.

### 3.3. Temporal Changes and Threat Factors

The broader environmental changes being driven by human activity and climate change can be expected in the region. However, their impact on charophyte habitats has not been described even at the national level and cannot be estimated without targeted research. Both the Sudzhuk Lagoon and Lake Sevan have seen documented negative changes in charophyte abundance and species richness [32,33,43,72,74,95,96,97,147], i.e., a decrease in the area of occupancy, maximal depth, and a reduction in belts to isolated stands. Negative trends could be expected in Azerbaijan in the area harboring rice fields in the middle of the 20th century. These water bodies are remarkable exclusions from the list of water bodies with charophyte records. However, comparing the data for different periods, it appears that neither severe decline nor the disappearance of charophyte sites and species richness can be detected at a large scale. We cannot indicate any case of this based on the specimens studied because no evidence for it is traceable in our dataset, except for the Sudzhuk Lagoon. Large changes, probably mostly negative ones, can be expected in the coastal area of the Caspian Sea because of large-scale water level changes, urbanization, and oil and gas production.

The sulfur spring near Alagir, Republic of North Ossetia—Alania, harboring a population of *Chara gymnophylla,* is a remarkable example of the long-term existence of a charophyte population for over a century. This species has been known here since 1901 (see above in the Results). The stable presence and good conditions of the charophyte populations in the lakes in the Goygol National Park in Azerbaijan can be estimated, from the scarce collections and published records, as dating from at least from the 19th century. Charophytes are still an important part of the aquatic vegetation of Lakes Sevan and Lisi, according to recent surveys [72,73,83], although some negative effects of artificial water level change could be expected for perennial species. The populations of large perennial species, like *C. globata*, *C. hispida*, *C. papillosa*, and *C. tomentosa,* could be the most stable charophyte sites, but the scarcity of surveys leaves little evidence for this suggestion. The same reason limits the elucidation of threat factors and important changes in particular water bodies and regions.

### 3.4. Further Perspectives

Merging and transforming all the available datasets for Caucasian charophytes into a single reliable one is a challenge for future studies. Specimen-based evidence appears to be a good solution, but sets of key-trait images for small populations of rare species would be helpful too. This research is a prerequisite for gaining more complete recent data, creating a reference point for tracking the changes in the abundance and occurrence of charophytes species in the Caucasus. The scarcity of recent observations prevents the estimation of species occurrence trends and the elucidation of threatening factors. These are essential for the preparation of national Red Lists. A survey of all the precisely geo-referenced sites and new ones would be desirable for the same aim. Checking specimens stored in national herbaria is essential for further updates to this dataset. The authors invite all colleagues interested in the aquatic macrophytes of this diverse region to contact us for joint research and consultations about Caucasian charophytes.

Important re-identifications could be expected for the dominant charophyte species in Lake Sevan. It is probable that some records of *Chara papillosa* (as *C. intermedia*) were based on *C. globata*, considering the difficulties in the delineation of these species based on plants growing at low insolation [55]. Some records of *C. globularis* appear to be based on *C. uzbekistanica*. Recent surveys of the aquatic vegetation of Lake Sevan could shed light on this uncertainty. Considering the wide variability in *C. contraria*, resulting in a partial to complete loss of the cortex and the uncertainty associated with the species of *Chara* having an imperfect cortication of branchlets and stem [39,54], it cannot be ruled out that records of *C. denudata* could be referring to it or *C. squamosa,* if more recent specimens will be available from its site. Re-collecting complete, well-developed, fertile charophytes will help to reduce uncertainty in all cases across the Caucasus.

## 4. Materials and Methods

This research focused on a mountain area, delimited with an approximate border between the mountain landscapes and the plain area northward and northwestward of the Caucasus, traceable on the map of relief and tentatively outlined with the northernmost sites of the species (Figure 1, Figure 2, Figure 3 and Figure 4). The southern border of the studied area is delimited by the state borders with the Republic of Türkiye and the Islamic Republic of Iran, because of the scarcity of charophyte records in their Caucasian areas [59,70,122,128].

New specimens were collected by hand or with a grapnel during a careful survey of water bodies. The coordinates of the collection sites, indicated in the labels of the studied specimens above and in Appendix A, were taken in the field. The specimens were dried as herbarium specimens and stored in the collections of the Komarov Botanical Institute of the Russian Academy of Sciences (LE), Omsk State University (OMSK), and the Papanin Institute of Inland Water Biology of the Russian Academy of Sciences (IBIW) (acronyms are according to Thiers [148]).

The collections of the Naturhistorisches Museum Wien (W), ZE Botanischer Garten und Botanisches Museum, Freie Universität Berlin (B), Naturalis Biodiversity Center (L), University of Helsinki (H), IBIW, and LE were checked. The specimens were identified by A. Braun, A. Petunnikow, J. Vilhelm, M.M. Hollerbach, L.K. Krassavina, T.V. Sviridenko, and F. Noedoost. However, some misidentifications were spotted, and updated identifications are listed above and in the Appendix. Since the 19th century, some specimens have been stored without any identification until this study. The morphological features of the specimens were studied using an Olympus SZ61 stereomicroscope (Olympus Corporation, Shinjuku, Tokyo, Japan). The identification was based on morphological traits widely used for charophytes [39], but the barcoding of some specimens from Dagestan was implemented earlier by us (see the description of methods and results in [14]). Neither cryptic nor new species were found in Dagestan, and all the charophyte accessions were resolved within the already known haplotypes [14]. The key for European charophytes [39] is largely useful for the species known from the Caucasus, because it covers all of them. The species concepts and criteria for species delineation described in detail in the book about European charophytes [39] were completely followed during this study.

Compiling the published records, scattered in many references and frequently inaccessible at the international level, was also important as an essential step for tracking changes in the occurrence of charophyte species. We compiled a list of 176 published geo-referenced species distribution records. The vouchers for 55 of them were checked. They are indicated on the maps as based on specimens (Figure 1, Figure 2, Figure 3 and Figure 4). The published geo-referenced species distribution records for which the vouchers have not been located yet, are indicated on the species distribution maps (Figure 1, Figure 2 and Figure 3), because we cannot confirm the identification of each published record.

This research summarizes all the data from the studied specimens and published records, both printed and from online resources [83,104] (Figure 1A), using only research-grade observations in iNaturalist. However, we grouped these records separately, hoping to the check records not supported by the studied specimens in the future. All the published records for *Chara* spp., *Nitella* spp., and unidentified charophytes [21,25,31,35,37,48,87,149,150,151] (and references within) are depicted on the map (Figure 4B) to outline the known distribution of Caucasian charophytes as completely as possible. The species distribution is illustrated in the maps prepared with SimpleMappr, version 1.0 [152]. The Jaccard index [153] was used for revealing the similarity of the charophyte floras. The species concepts, delineation between species, and nomenclature follows the latest reference [39], largely suitable for the Caucasus.

## Figures and Tables

**Table 1:** Species of charophytes in Caucasian countries according to different sources.

Species	Armenia			Azerbaijan			Georgia			Russia		
	PR	SP	ON	PR	SP	ON	PR	SP	ON	PR	SP	ON
*Chara baltica* (Hartm.) Bruz.	–	–	–	+	+	+	–	–	–	+	+	–
*Chara braunii* C.C. Gmelin	–	–	–	+	+	–	–	–	+	–	–	–
*Chara canescens* Desv. et Lois. in Lois.	+	+	–	+	+	+	+	–	+	–	+	+
*Chara connivens* Salzm. ex A. Braun	–	–	–	–	–	–	+	+	+	+	+	–
*Chara contraria* A. Braun ex Kütz.	+	+	–	+	+	–	+	+	+	+	+	–
*Chara denudata* A. Braun	–	–	–	+	+	–	–	–	–	–	–	–
*Chara globata* Migula	+	+	–	–	–	–	–	–	+	+	+	+
*Chara globularis* Thuill.	+	–	–	+	–	–	+	+	+	–	+	–
*Chara gymnophylla* A. Braun	+	+	+	+	–	–	+	+	–	+	+	–
*Chara hispida* L.	–	–	–	+	–	–	–	–	+	–	–	–
*Chara neglecta* Hollerbach	–	–	–	+	+	–	–	–	–	+	+	–
*Chara papillosa* Kütz.	+	+	–	+	+	–	+	+	+	–	+	–
*Chara squamosa* Desf.	–	–	–	–	–	–	–	+	–	+	+	–
*Chara strigosa* A. Braun	–	–	–	+	–	–	–	–	–	–	–	–
*Chara tomentosa* L.	+	–	–	–	–	–	–	–	–	+	–	–
*Chara uzbekistanica* Hollerbach	–	+	–	–	–	–	–	–	–	–	–	–
*Chara virgata* Kütz.	–	–	–	–	+	–	–	–	–	–	–	–
*Chara vulgaris* L.	+	+	+	+	+	+	+	+	+	+	+	+
*Lamprothamnium papulosum* (Wallr.) J. Groves	–	–	–	–	+	–	–	–	+	+	+	–
*Nitella capillaris* (Krock.) J. Groves et Bull.-Webst.	–	–	–	–	+	–	+	–	–	–	–	–
*Nitella flexilis* (L.) C. Agardh	–	–	–	–	–	–	–	–	–	–	+	–
*Nitella mucronata* (A. Braun) Miq.	–	–	–	–	–	–	–	+	+	–	+	+
*Nitella opaca* (C. Agardh ex Bruzelius) C. Agardh	–	–	–	–	–	–	–	–	+	–	–	–
*Nitellopsis obtusa* (Desvaux) J. Groves	–	–	–	–	–	–	–	–	+	–	–	–
*Sphaerochara prolifera* (Ziz ex A. Braun) Soulié-Märsche	–	–	–	–	–	–	+	–	–	–	–	–
*Tolypella glomerata* (Desv. in Loisel.) Leonh.	–	–	+	+	+	–	–	–	+	–	–	–
* Tolypella nidifica* (O.F.Müll.) A. Braun	–	–	–	–	–	–	–	–	–	+	+	–
Species number	8	7	3	13	12	3	9	8	14	11	15	4

Abbreviations: PR—published references; SP—studied specimens; ON—records from GBIF.org [104] and iNaturalist.org [83]. The records from the published references checked by the authors by specimens are indicated as studied specimens too.

## Data Availability

The dataset is available on request from the authors.

## Appendix A

The appendix contains a list of the studied specimens of the generalist species of Caucasian charophytes.

*Chara contraria* A. Braun ex Kütz. var. *contraria*

Material examined: Armenia • Vedi Region, Arazdayan [Yeraskh], ditch; 18 July 1954; Academia Scientiarum Arm. SSR. Hortus Botanicus Erevanensis; LE. • Azerbaijan • Masally District, the village of Gegechol [Giga-Chel], in istil [see explanation of this local name in the note for *C. contraria* var. *contraria* in Results]; 2 June 1958; D.A. Aliev leg.; LE: 144(1). • Khanlar District [Goygol District, Goygol National Park], M.K. Maralgel’ [Lake Maralgol]; 12 July 1970; F.A. Babaev leg.; LE. • Khanlar District [Goygol District, Goygol National Park], M.K. Shamlyggel’ [Lake Shamlygel, Lake Shamlygyol]; 13 July 1970; F.A. Babaev leg.; LE. • Khanlar District [Goygol District, Goygol National Park], M.K. Zeliligel’ [Lake Zeligol]; 16 July 1970; F.A. Babaev leg. (LE). • Georgia • Abkhazia. Loc. Skurcza [Skurcha] prope ostium fl. Kodor [Kodori River]. In aqua stagnante; 12 June 1904; G. Woronow leg. Plantae caucasicae, No. 121; LE. • [Abkhasia], Gagra, the road to the Cape Pitsunda, a pond among the fields to the right of the road before reaching Bzyb [Bzipi], in very shallow water, in clay; 23 May 1940; V.L. Komarov leg.; LE: A0001315, LE: A0001316. • Vicinity of Tbilisi, Lake Lisi-Tba [Lake Lisi], northern part; 13 June 1939; T.E. Dzhibladze leg.; LE: 41(1), LE: 41(2), LE: 41(3). • Tbilisi, local district Vake, Lake Cherepashye (Lake Kustba); 41.7004° N; 44.7527° E; 5 May 2017; A.N. Efremov leg.; LE. • Russia • Kabardino-Balkarian Republic, Lake Nizhnee Goluboe [Lake Cerik-Köl]; 26 June 2016; E.N. Patova leg.; LE. • Kabardino-Balkarian Republic, Lake Nizhnee Goluboe [Lake Cerik-Köl]; 5 January 2021; D.F. Afanasyev leg.; LE: A0000440.

*Chara gymnophylla* A. Braun

Material examined: Armenia • Geghark’unik’ mars, NE side of Lake Sevan, neben Pambak, humid, partly boggy meadow; 40.3819° N; 45.5261° E; alt: 1965 m a.s.l.; 4 July 2015; G. Fayvush, M. Oganesian, J. Koopman, H. Więcław, A. Aleksanyan, E. Vitek leg., 15-0259; W 2016-00325, W 2016-00326. • Lorri province, area c. 7.8 km W of Stepanavan, besides road c. 0.85 km S Urasar, *Typha* bog and dry gravel in surroundings; 41.0080°N; 44.2905°E; alt: 1525 m a.s.l.; 30 August 2012; G. Fayvush, M. Oganesian, J. Koopman, H. Więcław, A. Aleksanyan, E. Vitek leg., 15-0259; W 2014-04428, W 2014-04429. • Georgia • In stillstehendem Wasser am Aragwi bey Ananuri; Sept[ember], [before 1841]; [R.F.] Hohenacker, leg., 3761; LE. • In langsam fließendem Wasser und in Pfützen bey Hildt(?); Juny (?), [before 1841]; [R.F.] Hohenacker leg., 1831; LE. • In der Pschawischen Aragua **** [illegible letters] Magaro * [illegible letters] Orizchale, 500 hex.; 21 September 1860; [F.J.] Ruprecht leg.; LE: A0001288. • East South Caucasus, Yugo-Ossetia [South Ossetia], vicinity of the city of Stalinir [Tskhinvali, Tskhinval], small swamp in stream bank in the floodplain of Bol’shaya Liakhva River [Great Liakhvi River], 800 m a.s.l.; 18 August 1946; I. Abramov leg.; LE: 76(2). • Yugo-Ossetia [South Ossetia], Dzava District [Dzau District], in a stream near Elbakit [Yelbakita], at forest edge near the road, 1200 m a.s.l.; 5 August 1947; A. Abramova leg.; LE: 76(1). • Sagarejo District [Sagarejo Municipality], branch of Iory River [Iori River] near the village of Sioni, stands over bottom; 4 September 1951; T.I. Imermeshvili leg.; LE: 94(1), LE: 94(2). • East South Caucasus, Yugo-Ossetia [South Ossetia], Stalinir District [Tskhinvali District], the right slope of the valley of Malaya Liakhva River [Little Liakhvi River], small swamp below the settlement of Vanaty [Vanati, Uanat]; 12 September 1960; A. Abramova leg.; LE. • Russia • Reg. Tersk. [Stavropol Territory], Kislovodsk; 8 July 1889; I. Akinfiev leg.; LE; reported by Vilhelm [59] and Hollerbach [37] as *C. foetida* f. *longibracteata* (Kütz.) A. Braun, a synonym of *C. vulgaris* f. *longibracteata*. • Ossetia [Republic of North Ossetia–Alania], sulfur spring; 27 June 1901; [V.V.] Marcowitsch leg.; LE. Ossetia, sulfur spring of Voe. Os. Dor. [Ossetian Military Road]; 30 August 1901; [V.V.] Marcowitsch [Marcowitseh on the printed label] leg.; LE. • [Stavropol Territory], near Pyatigorsk, a branch of Podkumok River, in shallow weak running water; 29 August 1948; L.M. Zauer leg.; LE. • Krasnodar Territory, station of Makopse on the Black Sea; original label: mountain stream entering the Black Sea [Makopse River], station of Makopse (15 km south Tuapse), Holiday Center Zheleznodorozhniy, 1 km above stream mouth; 24 July 1955; E.N. Vaulina leg.; LE. • [Republic of North Ossetia–Alania] Alania near Alagir, in sulfur spring; 1 September 2012; D.[S.] Shilnikov leg.; LE. • Kabardino-Balkarian Republic, Chereksky District, village of Aushiger, stream in floodplain of Cherek River, depth 5–20 cm; 43.3791° N; 43.7284° E; 2 September 2021; O.N. Boldina leg.; LE: A0000997, LE: A0000998.

*Chara vulgaris* L. f. *vulgaris*

Material examined: Armenia • Vedi Region, Arazdayan [Yeraskh], wetland soils; 4 June 1955; Academia Scientiarum Arm. SSR. Hortus Botanicus Erevanensis; LE. • Razdan District [Hrazdan District, now Kotayk Province], Marmarnk River basin, left bank of the river, in the vicinity of the settlement of Ahavnadzor [Aghavnadzor], old riverbed, swampy area; 21 July 1983; N. Khandzhyan leg.; LE, ERA: 159847. • Azerbaijan • Gub[erniya] Baku, distr[ict] Kuba [Quba], prope Chaczmaz [Khachmaz, Xaçmaz], in oryzetis; 140′ [feet, ~43 m a s.l.]; 25 June 1899; Alexeenko leg., Flora Caucasi, No. 723; LE. • Gub[erniya] Bacu, distr[ict] Geokczai [Geokczai Uezd, Goychay, Göyçay], ad st[.] viae ferr. Műsűsli [Müsüslü], in fossis; 19 April 1902; Alexeenko leg., Flora Caucasi Ex. 17469a; LE: 93. • Gub[erniya] Bacu, distr[ict] Geokczai, inter p. Incza [Incha, İncə] et Alpaut [Alpout], in paludosis; 19 April 1902; Alexeenko leg., No. 92 unic., Flora Caucasi Ex. 17381; LE: 91. • Guberniya Baku, Lenkoran Uezd, slow-flowing water of the backwaters of the Lenkoranka River [Lənkərançay] near the village of Germatuk [Gərmətük]; 8 May 1916; N.N. Woronichin leg., Hortus Botanics Tifliensis; Herbarium cryptogamicum; LE. • Apsheron Peninsula, st[ation] Plyazh [Beach], wetlands; 12 May 1958; D.A. Aliev leg.; LE: 143(2), 143(3). • Apsheron Peninsula, st[ation] Plyazh [Beach], wetlands on the Caspian seashore; 21 May 1957; D.A. Aliev leg.; LE: 143(3). • Lenkoran District, the village of Sutamurdov [Lankaran District, Sütəmurdov (also, Sutamurdo and Sutamurdob)], in rice fields; 9 July 1956; D.A. Aliev leg.; LE: 137(4). • Lenkoran District, the village of Sutamurdov, in rice fields; 4 July 1956; D.A. Aliev leg.; LE: 137(2). • Lenkoran District, Island Sara, puddle-like freshwater bodies on the shore of the Bolshoy Kyzyl-Agach Bay separated from bay water by a ridge of shell rock and a strip of rushes; 7 February 1959; T.[N.] Kutova leg.; LE: 141(3). • Lenkoran District, Malyy Kyzyl-Agach Bay with fresh water, flood of River Kumbashinka; 7 February 1959; T. N. Kutova leg.; LE: 141(2). • Lenkoran District, the village of Diya [probably Digah], in rice fields; 7 June 1956; D.A. Aliev leg.; LE: 137(5). • Khanlar District [Goygol District, Goygol National Park], M.K. Gek-gel [Lake Goygol, Göygöl]; 11 July 1970; F.A. Babaev leg.; LE. • Georgia • [Mtskheta-Mtianeti region], prope pagun Kasbek [near the village of Kazbegi, now Stepantsminda]; alt. 900 hexap. [~274 m a.s.l.]; 2 August 1844; Dr. Kolenati leg., N. Supplementum 1. (unicum) Chara. Flora Caucasica; LE. • Vicinity of Tbilisi, Lake Lisis-Tba [Lake Lisi], northern part; 13 June 1939; T.E. Dzhibladze leg.; LE. • Yugo-Ossetia [South Ossetia], Dzhav District, Ermani area, Kom-Komme tract, Lake Dzoar-Shad [probably Lake Tsilgidzuar, Lake Tsilghidzwar]; 2550 m a.s.l.; 21 September 1947; I. Abramov leg.; LE: 76(3). • East South Caucasus, Yugo-Ossetia [South Ossetia], Stalinir District [Tskhinvali District], the right slope of the valley of Malaya Liakhva River [Little Liakhvi River], small swamp below the settlement of Vanaty [Vanati, Uanat]; 12 September 1960; A. Abramova leg.; LE. • South Ossetian Autonomous Oblast, Stalinir District [Tskhinvali District], a stream along the bed of the Khevi ravine near the village of Vanata; 14 September 1960; A.L. Abramova, I.I. Abramov leg.; LE. • Abkhaz ASSR, vicinity of Sukhumi, stream near the village of Verkhnie Merkheuli [Merkheuli]; 31 August 1963; A.L. Abramova, I.I. Abramov leg.; LE. • Tbilisi, Tbilisi Botanical Garden; 10 May 2014; A.N. Efremov leg.; LE, OMSK. • Abkhazia, the settlement of Tsandrypsh [Gantiadi], between Khashpsy River (Khashupsa) and railway station, the territory of a former concrete factory near the seashore, puddle; 43.3742° N, 40.0888° E; 23 July 2023; G.Yu. Konechnaya leg.; LE. • Russia • Krasnodar Territory, Black Sea District, vicinity of Novorossiysk; 7 May 1892; S. Lipsky leg.; LE: A0001439. • [Stavropol Territory] Caucasus, Zhelezhnovodsk; 30 July 1894; I. Akinfiev leg.; LE: A0001286. • Kabardino-Balkaria, the Chegem river basin, on the way to the Chegem Gorge from Nalchik, a puddle by the road behind the village of Lechinkay [Lichimkay in the diary of Hollerbach]; 16 August 1951; M.M. Hollerbach leg., No. 178 bis, Central Caucasian Expedition 1951; LE. • Dagestan and the Chechen Republic, Lake Forelnoe (mountain) [Lake Kezenoyam]; 12 August 1968; V.M. Katanskaya leg.; LE. • Krasnodar Territory, Apsheronsky District, 3 km south of the Samurskaya Station, stagnant water; 12 August 2004; S.V. Bondarenko leg.; LE. • Krasnodar Territory, Arkhipo-Osipovka, stream near the sea; 19 April 2014; G.Yu. Konechnaya leg.; LE. • Chechen Republic, Kurchaloevsky District, the village of Kurchaloy, Khumyk River near the bridge; 43.2025° N; 46.1079° E; 25 June 2018; G.Yu. Konechnaya leg.; LE: A0000968.

*Chara vulgaris* f. *longibracteata* (Kütz.) H. Groves

Material examined: Azerbaijan • Baku Guberniya, Lenkoran; 8 May 1893; V. Lipsky leg.; LE: A0001287. • Gub[erniya] Baku, distr[ict] Kuba [Quba], ad fl. Ata-czai prope p. Bakschali, in stagnates; 2500′ [feet, ~762 m a.s.l.]; 24 July 1900; Alexeenko leg., Flora Caucasi, No. 5852; LE: 113. • Gub[erniya] Baku, distr[ict] Schemacha [Shemakha Uezd], [inter] p. Marazy [Mərəzə, Qobustan], ad rivulum “Solenyi Fontan”; 2400′ [feet, ~732 m a.s.l.]; 2 September 1900; Alexeenko leg., Flora Caucasi, No. 10229; LE: 115. • Gub[erniya] Baku, distr[ict] Geokczai [Geokczai Uezd, Goychay, Göyçay], inter p. Incza [Incha, İncə] et Alpaut [Alpout], in paludosis; 19 June 1902; Alexeenko leg.; LE. • Kuba Uezd [Quba District], between [railway] stations Khudat and Kusar-chay, river; 19 July 1927; I.I. Karyagin leg.; LE: A0001479. • Apsheron Peninsula, the railway station of Zagul’ba, small water body; 9 May 1958; D.A. Aliev leg.; LE: 137(1); LE: 143(1). • Georgia • Abkhazia, Khashupsa River near mineral spring; 43.3958° N; 40.1047° E; 30 August 2011; G.Yu. Konechnaya leg.; LE. • Russia • Krasnodar Territory, Sochi, Adler District, Imereti Lowland, irrigation system, Black Sea coast, near the mouth of Psou River, canal; 3 July 2008; A.N. Efremov, B.F. Sviridenko leg.; LE, IBIW. • Krasnodar Territory, the vicinity of the village of Shirokaya Shchel, Sheps River, a heated flowing backwater; 44.5756°N; 38.1684°E; alt: 20 m a.s.l.; 5 July 2018; D.G. Melnikov, A.V. Popovich leg., No. 209a; LE. • Krasnodar Territory, vicinity of the settlement of Aderba; 44.5541° N; 38.1431° E; alt: 38 m a.s.l.; 5 July 2018; D.G. Melnikov, A.V. Popovich leg., No. 220; LE. • Krasnodar Territory, the city of Khedyzhensk, a puddle in the road above the sanatorium “Mineralniy”; 14 July 2021; E.P. Saranchin leg.; LE: A0000458.
